# Bad Temper and Insanity

**Published:** 1874-03

**Authors:** 


### BAD TEMPER AND INSANITY.

Passionate people, the hasty kind, who flare up in a blaze, like fire to tow, or a coal to powder, without taking time to inquire whether there is any ground for such a pyrotechnic display, and then get more furious, when they find out there was no cause for their fiery feats, may learn a useful, as well as a serious lesson from an item in Dr. Blanchard's report of the King's County Lunatic Asylum, that "*three men and three women became insane by uncontrollable temper.*"

We all feel a sympathy for one who has become demented from loss of kindred, from disappointment, and from a hard lot in life; but we can have no such feeling for quarrelsome, ill natured, fretful, fault finding, complaining, grumbling creatures, the greater part of whose every day life tends to make those whose calamity it is to be bound to them, as miserable as themselves. I consider ill nature a crime, and like other crimes, is ordained in the government of God, to meet, sooner or later, its merited reward. Other vile passions may have some points of extenuation, the pleasure for example, which may attend their indulgence, but ill nature, that is, a fretful, fault finding spirit, in its origin, action and end, has no extenuating quality; and in the application of the Scripture principle, "*with what measure ye mete, it shall be measured to you again,*" will find a pitiable end. Therefore, with all the power God hath given you, strive, reader, and strive for life, to *mortify* this *deed of the flesh.* Watch hourly, watch every moment against the indulgence of a hasty temper as being offensive to your Maker, and contemptible in the eyes of your fellow man ; contemptible, because for the person who possesses it, and knows it, yet indulges in it, and makes no effective efforts to restrain it, no human being can have any abiding attachment or respect, founded as it is, in low morals, or low intellect, or both.

